# Supplementary material for: Temporary Employment Is Associated with Poor Dietary Quality in Middle-Aged Workers in Korea: A Nationwide Study Based on the Korean Healthy Eating Index, 2013–2021
Source: Nutrients. 2024 May 14;16(10):1482. doi: 10.3390/nu16101482 (PMC11124147; doi:10.3390/nu16101482)
Supplement: Supplementary file 1 [file nutrients-16-01482-s001.zip › nutrients-2927302-supplementary.pdf]

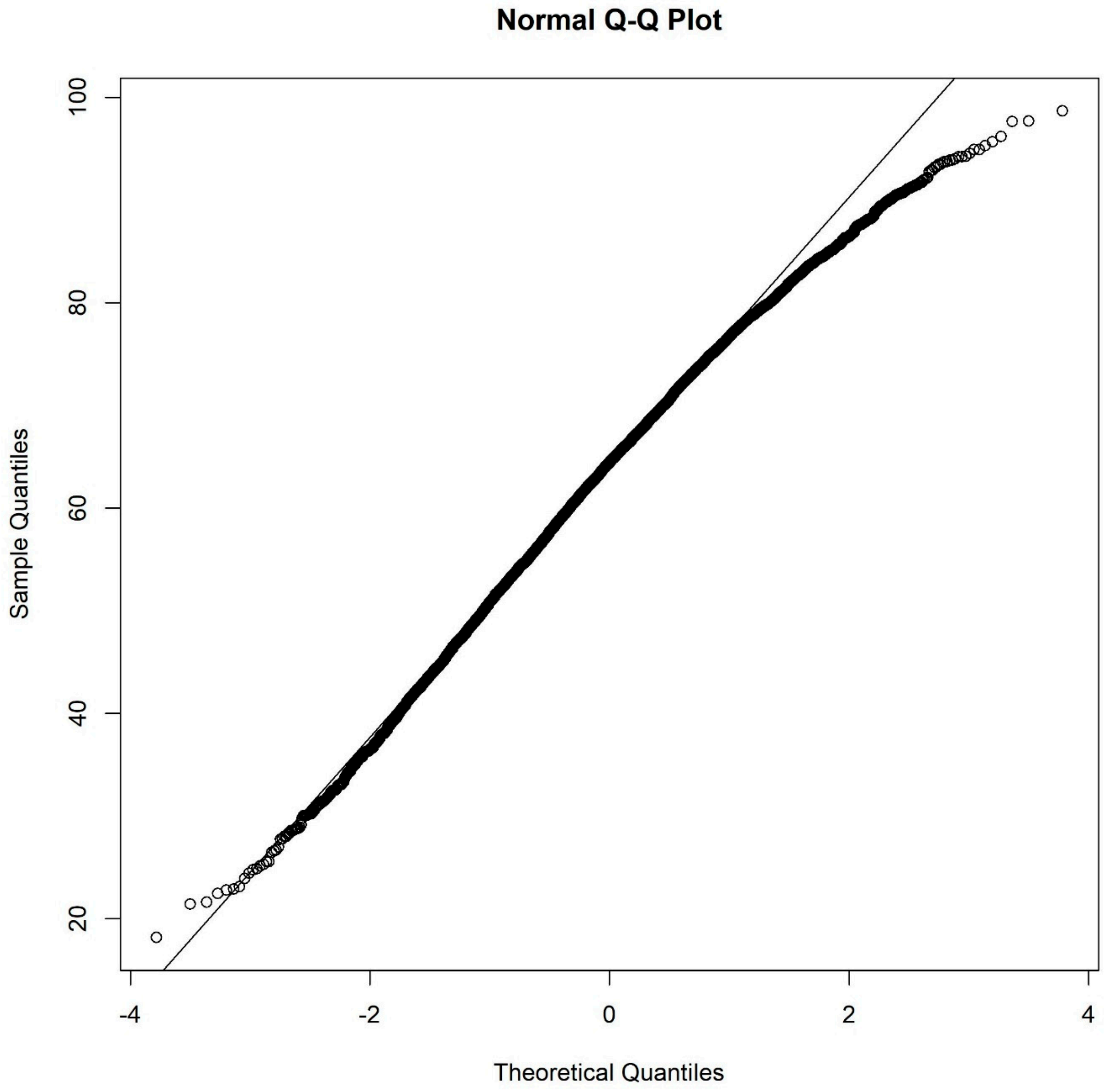

Figure S1. Q-Q plot of the distribution of Korean Healthy Eating Index.

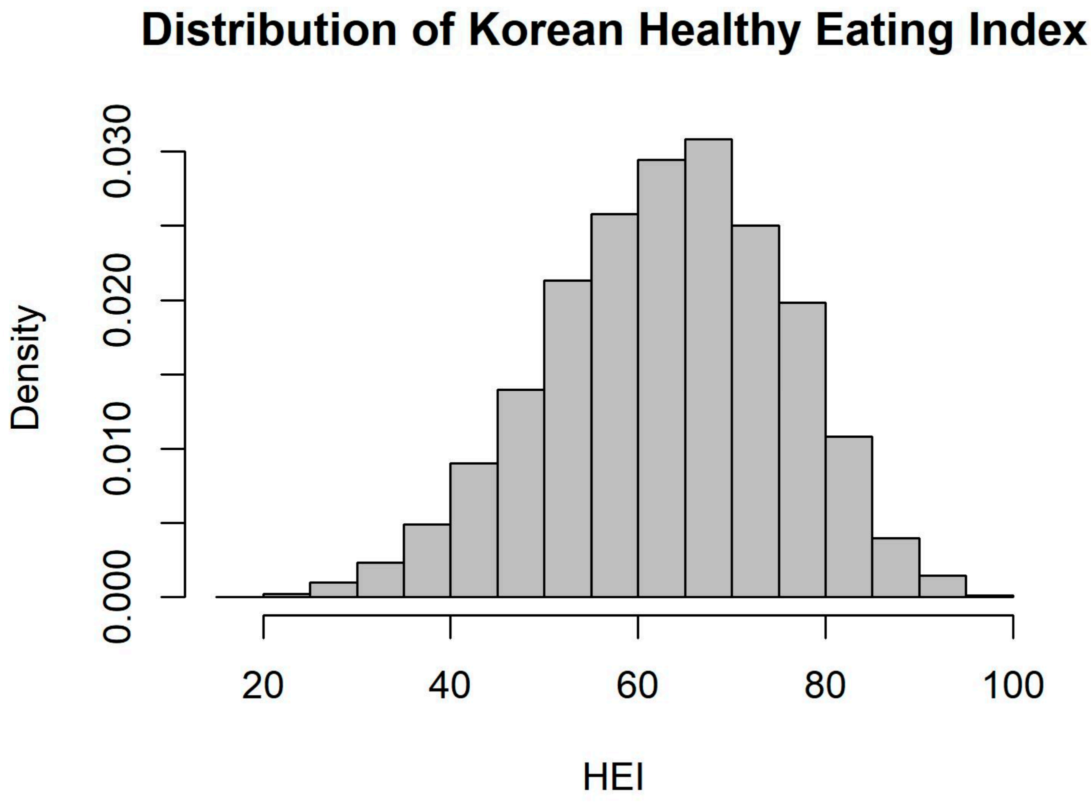

Figure S2. Histogram of the distribution of Korean Healthy Eating Index.

Table S1. Associations between temporary employment and the Korean Healthy Eating Index according to the souse's employment status.

	Men		Women	
	Working spouse	Non-working spouse or no spouse	Working spouse	Non-working spouse or no spouse
	β (95% CI)	β (95% CI)	β (95% CI)	β (95% CI)
**Employment type**				
Regular	0.00 (0.00, 0.00)	0.00 (0.00, 0.00)	0.00 (0.00, 0.00)	0.00 (0.00, 0.00)
Fixed-term	-0.38 (-3.20, 2.45)	-5.16 (-8.38, -1.95)	-0.72 (-2.06, 0.62)	-2.20 (-4.58, 0.19)
Daily	-3.26 (-6.28, -0.24)	-3.40 (-6.43, -0.37)	0.04 (-2.10, 2.19)	-5.80 (-9.73, -1.86)

β, beta coefficient; CI, confidence interval

The adjusted model controlled for age, education, income, occupation type, working hours, and survey years.
